# Mechanism of abscisic acid in promoting softening of postharvest ‘Docteur Jules Guyot’ pear (*Pyrus communis* L.)

**DOI:** 10.3389/fpls.2024.1502623

**Published:** 2024-12-17

**Authors:** Xiaofei Xu, Xinxin Zhu, Fudong Jiang, Qingyu Li, Aidi Zhang, Hongxia Zhang, Jianzhao Li

**Affiliations:** ^1^ The Engineering Research Institute of Agriculture and Forestry, Ludong University, Yantai, China; ^2^ Yantai Academy of Agricultural Sciences, Yantai, China; ^3^ School of Food Engineering, Ludong University, Yantai, China; ^4^ Shandong Institute of Sericulture, Shandong Academy of Agricultural Sciences, Yantai, China; ^5^ Zhaoyuan Shenghui Agricultural Technology Development Co., Ltd, Zhaoyuan, Shandong, China

**Keywords:** ‘Docteur Jules Guyot’ pear, abscisic acid, pectin, softening, postharvest

## Abstract

Abscisic acid (ABA) is a key hormone in plant growth and development, playing a central role in responses to various biotic and abiotic stresses as well as in fruit ripening. The present study examined the impact of ABA and nordihydroguaiaretic acid (NDGA) on various postharvest ‘Docteur Jules Guyot’ pear fruit characteristics, including firmness, pectinase activity, pectin content, volatile aromatic substances, and the expression of correlated genes. The results showed that ABA quickly reduced fruit firmness, increasing the activity of pectin degradation-related enzymes. The contents of water-soluble pectin (WSP) and ionic-soluble pectin (ISP) increased, and covalent binding pectin (CBP) decreased under ABA treatment. Among the detected volatile aromatic substances, the highest-level substance of the fruit was ester, and the ABA treatment significantly promoted the amount of ester substances. The cell wall disassembly-related genes *PcPME3*, *PcPG1*, *PcPG2*, *PcPL*, *PcARF2*, and *PcGAL1*, as well as ABA biosynthesis-related genes *PcNCED1* and *PcNCED2*, were also significantly induced by ABA. Conversely, all these genes were repressed in the NDGA treatment group. Therefore, it was speculated that ABA may promote the softening of postharvest European pear fruit by affecting the activity of pectin degradation enzymes in fruit cell walls.

## 1 Introduction

Pears can be processed in various ways, including canning the fruit or producing juice or syrup. They are rich in minerals and contain numerous bioactive compounds with anti-diabetic, anti-inflammatory, antibacterial, and anti-cancer properties (Öztürk et al., 2015). The ‘Docteur Jules Guyot’ pear (*Pyrus communis* L.) is a prominent European pear variety. This variety was introduced to China over a century ago and is primarily grown in the warm and semi-humid region of the Jiaodong area in Shandong province. Consumers favor European pears for their delicate taste and high nutritional value (Hewitt et al., 2020). However, European pears cannot be consumed immediately after picking and need to undergo ripening and softening to obtain a good taste.

During fruit ripening, chlorophyll in the pericarp is gradually broken down, while lutein and carotene in the chloroplast breaks down more slowly, so the pericarp appears red, orange, and yellow. It was discovered that the involvement of abscisic acid (ABA) significantly contributed to the enhancement of peel color modification. Anthocyanin accounts for a large percentage of the total antioxidant content in climacteric fruit figs, and it was found that ABA initiated anthocyanin biosynthesis and improved the color of figs (Lama et al., 2020). For non-climacteric fruit sweet cherry, the upregulation of *PavNCED1* and genes related to the ABA signaling pathway was observed during fruit transition from the light green stage to the pink stage (Kuhn et al., 2013). Pre-harvest application of S-ABA enhanced the development of a deep orange fruit color in early maturing M7 sweet orange varieties (Rehman et al., 2018). These findings indicate that the involvement of ABA is pivotal in the fruit ripening process, specifically in facilitating color change.

Alterations in cell wall composition led to increased tenderness in the fruit’s texture. The pectin-degrading enzyme activity influenced the change of pectin content in the cell wall. For climacteric fruit blueberry, the content of water-soluble pectin (WSP) in the ABA-treated group is higher than both the control group and nordihydroguaiaretic acid (NDGA)-treated group, and the pectin content of sodium carbonate soluble pectin (NSP), enzyme-soluble pectin (ESP), cellulose (CEL), and hemicellulose (HCEL), respectively, is lower than that in the control group and NDGA-treated group (Zhou et al., 2021). In the comparison of ‘Jingbai’ and ‘Yali’ pears, it was discovered that the former had a quicker softening rate, a higher pectin solubility, a much lower NSP content, and a significantly higher WSP content, whereas ‘Yali’ pears had a firm texture, and the pectin components were slightly changed during storage (Wei et al., 2015).

Besides the alterations in physiology, the volatile aromatic substances emitted by the fruit can also reflect the ripeness and overall quality throughout the maturation process (Obenland et al., 2012). Most of the aroma compounds in fruit accumulate during the early stages of ripening and reach their peak levels during the ripening stage (Han et al., 2022). The presence of aroma significantly influences the final sensory quality and fruit preferences among consumers (El Hadi et al., 2013). The aroma of pear varieties was attributed to esters, aldehydes, alcohols, ketones, and hydrocarbons, which are responsible for the aroma of different pears. These compounds have been extensively researched, with esters being the most significant (Willner et al., 2013). When kiwifruit was stored at low temperatures, the exogenous ABA treatment could regulate the synthesis of aroma compounds, and the considerable increase in esters’ level was also connected with this ripening phase time (Han et al., 2022). It was found that the synthesis of volatile esters can improve the flavor of bananas (Mukherjee et al., 2022). During the ripening process of the ‘Nanguo’ pear, the levels of esters increase while aldehydes decrease, leading to the grassy aroma of the fruit which gradually declines, allowing its unique aroma to emerge (Li et al., 2012).

ABA plays a role in controlling ethylene production and signaling to trigger the ripening of banana fruit (Thakur et al., 2019). In climacteric fruits such as peach and kiwifruit and in non-climacteric fruits such as grape, ABA was closely associated with the ripening and softening of the fruit, not only promoting the fruit coloration but also participating in the ripening of the fruit by affecting the activity of pectin degradation enzymes, such as polygalacturonase (PG), pectate lyase (PL), pectin methylesterase (PME), and cellulase (Zhang et al., 2009b; Gambetta et al., 2010; Fullerton et al., 2020). In mango, the PG activity was increased after ABA treatment, which resulted in the softening of the fruit (Zaharah et al., 2013). Ethylene started to stimulate PL expression in the early stages of banana ripening, and even after full ripening, the banana continued to express PL at a high level (Pua et al., 2001). Certain climacteric fruits, such as tomatoes, peaches, and avocados, all have high PG activities that correlate with the fruit softening rate (Brummell and Harpster, 2001).

ABA facilitates fruit ripening by stimulating the activation of genes associated with fruit softening. In peach fruit, virus-induced gene silencing of *PpPG21* and *PpPG22* downregulated the expression of these genes to affect the solubilization and depolymerization of peach pectin to achieve the purpose of delaying fruit softening (Qian et al., 2021). In persimmons, ABA can induce the expression of *DkACS2* and *DkACO1*, promote ethylene synthesis, and accelerate fruit softening, while NDGA delays their expression (Duan et al., 2015). ABA biosynthesis and catabolism in fruit cells involve both feed-forward and feedback pathways. The overexpression of *SlNCED1* can increase the endogenous ABA content, and the silencing of this gene can inhibit epidermal coloring and softening in tomato fruit (Taylor et al., 2000; Zhang et al., 2009a). *CYP707As* can inhibit the synthesis of ABA, and the *SlCYP707A1*, *SlCYP707A2*, and pear *PpcYP707A1* genes in tomatoes are engaged in the metabolic process of ABA during fruit ripening (Nitsch et al., 2009; Dai et al., 2014; Ji et al., 2014). For the non-climacteric fruit strawberry, feed-forward and feedback are closely associated with promoting *FveNCED* expression and inhibiting *FveCYP707A4a* expression (Liao et al., 2018).

The ‘Docteur Jules Guyot’ pear cannot be eaten immediately after picking. After softening, the storage time is greatly reduced, and the quality of the fruit decreases. Therefore, elucidating the role of ABA in fruit softening is crucial for promoting and extending the fruit’s shelf-life. To the best of our knowledge, the molecular mechanism underlying the softening process of ‘Docteur Jules Guyot’ pear fruit induced by ABA was limited. This research investigated the impact of ABA and NDGA on the ‘Docteur Jules Guyot’ pear, determining the fruit firmness, activity of pectin degradation-related enzymes, content of substance, and aroma compounds, thus illuminating the role of ABA in the postharvest ‘Docteur Jules Guyot’ pear softening.

## 2 Materials and methods

### 2.1 Plant materials

The fruit of ‘Docteur Jules Guyot’ pear was harvested in July 2022 from Yantai in Shandong Province. The fruits were picked at the commercial maturity stage, characterized by their uniform size and being free from mechanical damage, diseases, and pests, and were immediately transported to the laboratory. The fruits were treated with 15 L of 100 μM abscisic acid (ABA) and 100 μM nordihydroguaiaretic acid (NDGA) solutions for 30 min. In order to control the variables, the control group was also treated with water plus 50 mL ethanol and removed for drying after 30 min. Both the hormone-treated group and the control group pear fruits were stored at room temperature of 24°C to 25°C, with 16 days of storage. Every 4 days, samples were collected, and nine pear fruits were chosen randomly for sampling and index calculation from each group. Each experimental group was set with three biological replicates and each biological replicate with three fruits. After sampling the pear fruits, the pulp was retained, cut into pieces, and frozen with liquid nitrogen; then it was stored at -80°C for subsequent experimental research.

### 2.2 Quality determination

To measure the firmness of the fruit, a GY-4 fruit firmness tester with an 8-mm probe was used; the penetration depth was 1 cm, and the firmness was expressed by peak force (N). Using an ATAGO hand-held saccharimeter, the soluble solids and titratable acidity content of the fruit were measured. The L*, a*, and b* values were measured by using a Keshengxing CR-400 color colorimeter, which were expressed as positive and negative numbers.

### 2.3 Extraction of pectin from fruit

The extraction of pectin was based on the reference methods with some appropriate modifications (Zhang et al., 2018; Li et al., 2023). A 5-g pear fruit sample was weighed and mixed with 20 mL of 80% ethanol. After mixing well, the mixtures were placed in boiling water for 20 min and then centrifuged at 3,900 rpm for 10 min. Afterwards, the supernatant liquid was removed and disposed of, followed by rinsing and precipitating first with 6 mL of 80% ethanol, then with a chloroform/methanol mixture, and finally with acetone. The mixture was centrifuged at 3,900 rpm for 10 min, and the precipitate obtained after centrifugation was dried at 40°C to obtain the cell wall material (CWM). Furthermore, 50 mg of CWM was weighed, and 50 mM acetic acid–sodium acetate buffer (pH = 6.5), 50 mM acetic acid–sodium acetate buffer (pH = 6.5, containing 50 mM EDTA), and 50 mM Na_2_CO_3_ were sequentially added to extract water-soluble pectin (WSP), ionic-soluble pectin (ISP), and covalently bound pectin (CBP), respectively.

### 2.4 Activity of pectin degradation enzymes within the cell wall

All samples were removed from the -80°C refrigerator and stored in liquid nitrogen throughout the process before adding the solution. The activity of polygalacturonase (PG) was determined by the modified method of Gross (1982). Moreover, 3 g of sample was weighed, and 1.5 mL of 0.3 M NaCl was added and mixed, followed by centrifugation at 4°C and 10,000 *g* for 15 min. A control group was set up in addition to the assay group. The absorption value at 276 nm was recorded. The PG activity unit was expressed as the amount of enzyme required to convert 1 μmol of galacturonic acid per gram of FW per minute in the assay.

Pectate lyase (PL) activity was determined according to the modified method of Chourasia et al (2006). Samples of 0.25 g were weighed with 1.25 mL of 50 mM Tris-HCl (pH = 8.5, 0.6 mM CaCl_2_, 5 mM EDTA, and 0.5 mL Triton X-100), mixed, and centrifuged at 4°C and 10,000 *g* for 15 min. A 0.5-mL supernatant was absorbed, and 5 mL of Tris-HCl (0.6 mM CaCl_2_ and 2.4 g/L polygalacturonic acid) was added, and the initial absorption value at 235 nm was measured. The sample was then incubated in a water bath at 37°C for 30 min, and the absorption value was measured again. The PL activity unit was expressed as the amount of enzyme required to convert 1 μmol of unsaturated product per gram of FW per minute in the assay.

The activity of pectin methylesterase (PME) was determined according to Basak and Ramaswamy (1996). In detail, 1 mL of 0.3 M NaCl was added to a 0.8-g sample, and the mixture was centrifuged for 15 min under the same conditions, and 1 mL of supernatant was adjusted to pH = 7.8 with 0.05 M NaOH. A 0.5-mL aliquot was extracted, and 4.5 mL of 5 g/L citrus pectin solution was added, followed by adjusting the pH to 8.1 using NaOH of the same concentration. The centrifuge tube was placed in a 37-°C water bath for 20 min, with the pH adjusted to 8.1 using NaOH of the same concentration as needed. The volume of NaOH consumed to adjust the pH after holding at 37°C was recorded. The centrifuge tube containing the solution was then placed in water at 37°C, and the pH was adjusted to 8.1 with NaOH every 20 min. The amount of NaOH volume was recorded. An ice bath was used to preserve the enzyme activity during the assays. The PME activity unit was expressed as the amount of enzyme required to consume 1 μmol of NaOH per minute per gram of FW in the assay.

### 2.5 Determination of volatile aromatic substances

Headspace solid-phase micro-extraction method was used to extract volatile aromatic substances from the pear fruit sample. The determination of volatile aromatic substances was based on Zhang et al (2020) method. In detail, 2.0 g of sample was weighed, 3 mL of saturated NaCl solution was added, and 5 μL 2-octanol (0.8 mg/mL) was used as the internal standard. After placing the rotor into the bottle, the bottle’s opening was sealed with aluminum foil. The lid was secured, and the bottle was placed on a magnetic stirrer set to 45°C and at a speed of 260 rpm, with stirring for 30 min. A needle with an adsorption probe was inserted through the aluminum foil, the probe was extended into the liquid, and it was ensured that it did not touch the liquid beneath. Adsorption was conducted for 30 min, and the needle was inserted into a Shimadzu GC-2030 machine coupled with a Shimadzu GCMS-QP2020 NX (Shimadzu Co., Ltd., Kyoto, Japan) machine to determine the aromatic substances of fruits. The calculation of volatile aromatic substances was obtained by comparing the electron ionization mass spectral value measured by the sample with the NIST-2017 mass spectral library. Each sample in the experiment underwent three biological replicates.

### 2.6 RNA extraction and synthesis of cDNA

Total RNA was extracted from pear samples (0.5 g) for different storage periods using the total RNA isolation kit (Tiangen, Beijing, Chain). After determining the concentration of the extracted RNA using a microspectrophotometer, the RNA was reverse-transcribed to cDNA using the HIScript II Q-RT Super Mix for qPCR (+gDNA wiper) (Vazyme, Nanjing, China).

### 2.7 Expression analysis of relevant genes

Gene-specific primers were designed by using Primer 3 (http://bioinfo.ut.ee/primer3–0.4.0), listed in **Supplementary Table S1**. The RT-qPCR system was prepared by combining the ChamQ Universal SYBR qPCR Master Mix (Vazyme, Nanjing, Chain) with cDNA and gene-specific primers. Using a 0.1-mL 96-well PCR plate (CELLPRO, Suzhou, China) placed in a real-time PCR (Bio-Rad CFX ConnectTM Optics Module), the expression of different genes in a RT-qPCR system were analyzed.

### 2.8 Statistics and significant difference analysis

IBM SPSS Statistics 27 version was used to analyze the data. The single-factor ANOVA test method was used to analyze the significant differences among the groups. *P <*0.05 indicated significant differences between the data, and samples from different sampling points were repeated three biological times.

## 3 Results

### 3.1 Phenotype of ABA- and NDGA-treated pear fruit

**Figure 1:**
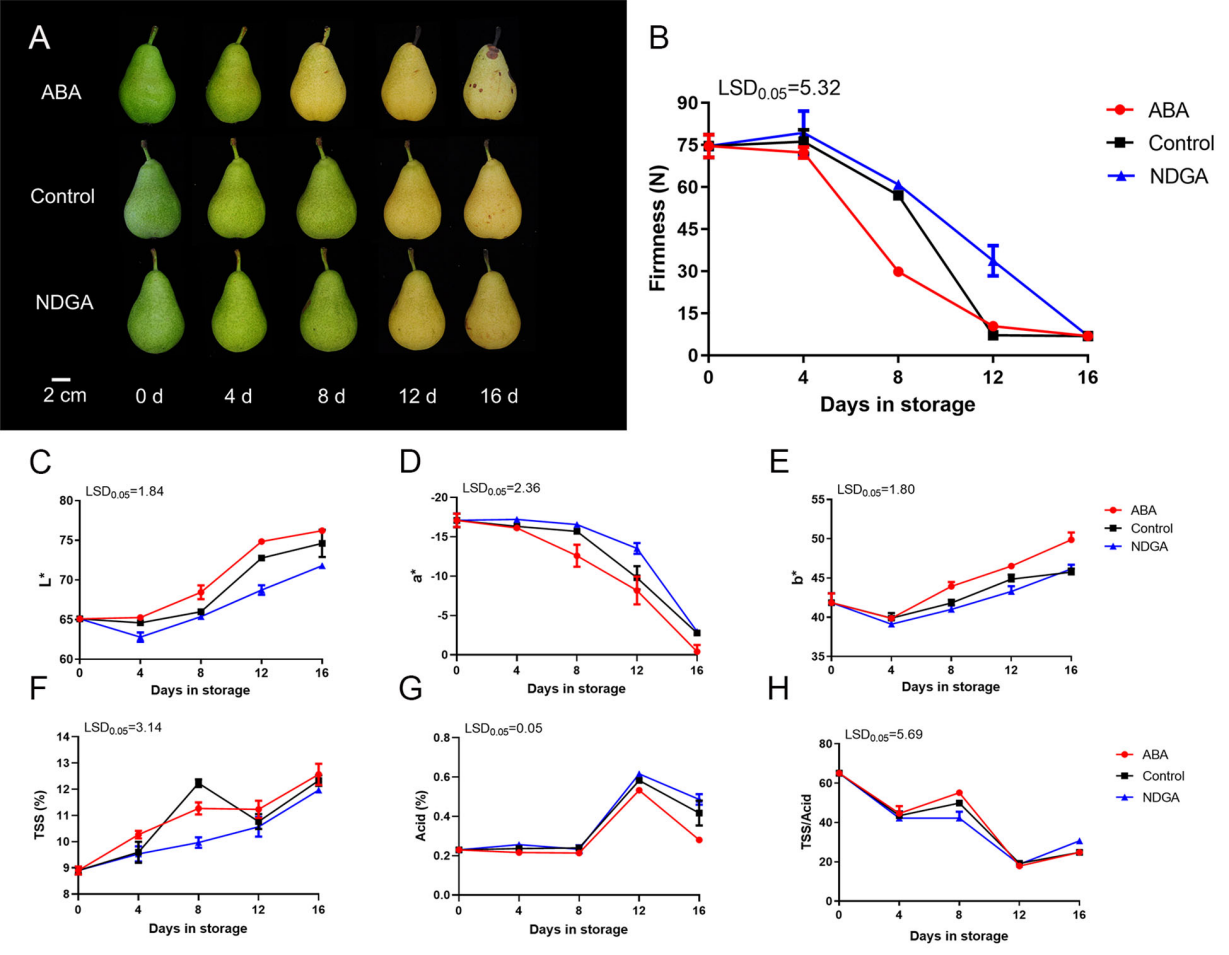
Phenotypes and physiological changes of ‘Docteur Jules Guyot’ pear after treatment with ABA or NDGA. **(A)** Appearance of ‘Docteur Jules Guyot’ pear. **(B)** Firmness of ‘Docteur Jules Guyot’ pear. **(C–E)** L*, a*, and b* of ‘Docteur Jules Guyot’ pear. L*, lightness; a*, reddish green degree (+: red, -: green); b*, yellowish blue degree (+: yellow, -: blue). **(F–H)** Total soluble solids (TSS) content, total acid content, and TSS/acid. The error bars come from three repeated SE. The observed LSD values suggest the presence of LSD with a significance level of *P* = 0.05.

The abscisic acid (ABA) treatment ‘Docteur Jules Guyot’ pear turned yellow early after 8 days, while the control group and nordihydroguaiaretic acid (NDGA) treatment group started turning yellow after 12 days (**Figure 1A**). The fruit firmness was initially 73.2 N, but under ABA treatment, the fruit firmness decreased rapidly, especially between days 4 and 8, from 72.2 to 29.8 N. Comparatively, the control group and the NDGA treatment group softened rapidly between days 8 and 12 from 57.1 to 7.1 N and 60.8 to 33.7 N, respectively (**Figure 1B**). By evaluating the color of the fruits using a colorimeter, the lightness (L*) of the control group was lower than that in the ABA-treated group. In the red–green degree of the fruit (a*), the green color of the ABA-treated group was inhibited, and the NDGA treatment group better maintained the green color of the fruit, while in the yellow–blue degree (b*), the ABA treatment group exhibited higher yellow values (**Figures 1C–E**). The general trend of TSS increased as the fruits continued to ripen (**Figure 1F**). Acid grew starting on day 8, peaked on day 12, and subsequently declined (**Figure 1G**). Furthermore, the TSS/acid ratio also dropped during storage (**Figure 1H**).

### 3.2 Changes in the content of pectin substances and the activity of pectin degradation enzymes

**Figure 2:**
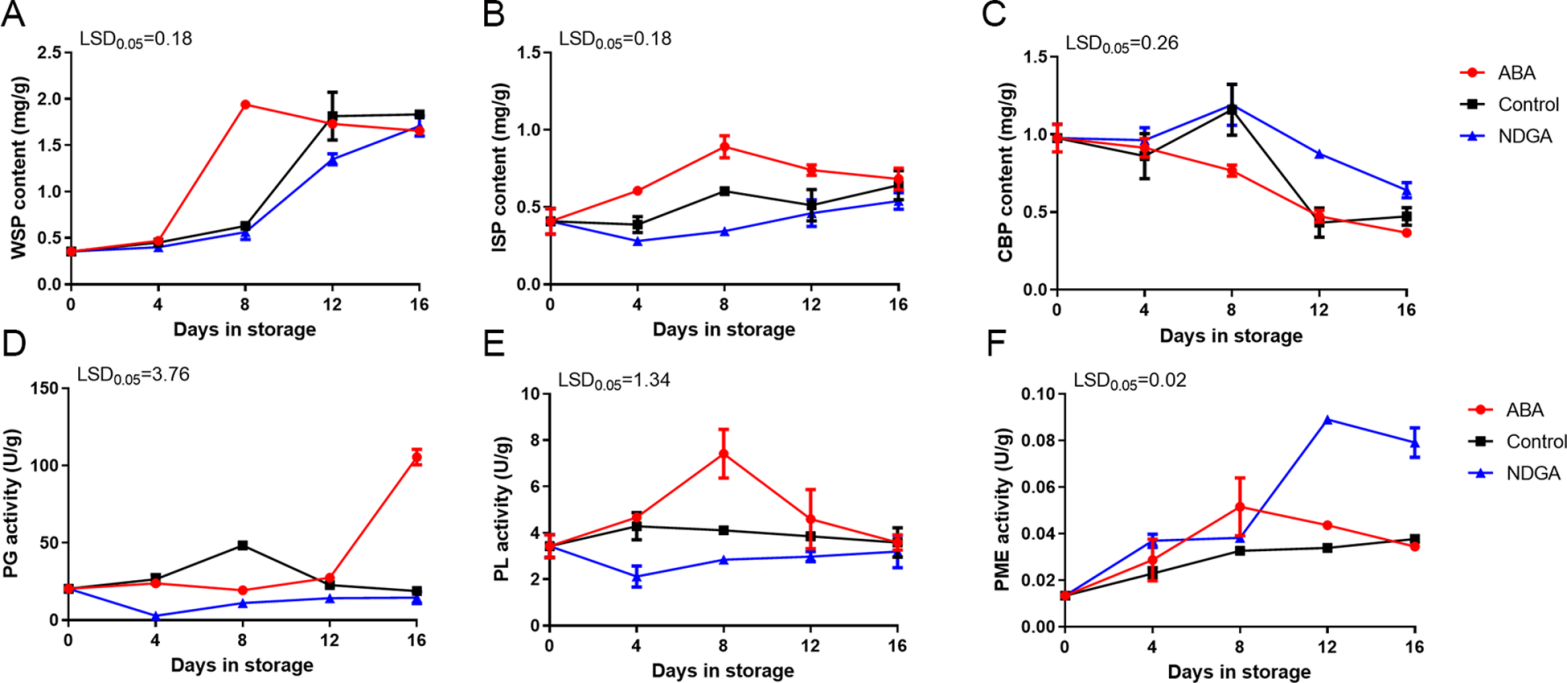
Pectin content and pectin degradation enzymes of ‘Docteur Jules Guyot’ pear after ABA or NDGA treatment. **(A)** Content of water-soluble pectin (WSP). **(B)** Content of ionic-soluble pectin (ISP). **(C)** Content of covalent binding pectin (CBP). **(D)** Activity of polygalacturonase (PG). **(E)** Activity of pectin methylesterase (PME). **(F)** Activity of pectate lyase (PL). The error bars come from three repeated SE. The observed LSD values suggest the presence of LSD with a significance level of *P* = 0.05.

The water-soluble pectin (WSP) content showed an overall increasing tendency during the postharvest storage, particularly under ABA treatment, which peaked on day 8. In contrast, the control group and the NDGA treatment group reached the maximum on days 12 and 16 (**Figure 2A**). The content of ionic-soluble pectin (ISP) increased and then decreased, and in the ABA treatment, the content of ISP was more than the control and the NDGA treatment groups (**Figure 2B**). The covalent binding pectin (CBP) content decreased sharply under ABA treatment, while the control group and the NDGA treatment group displayed a tendency of initial increase followed by a decrease (**Figure 2C**). Before 12 days, the activity of polygalacturonase (PG) was comparatively steady and increased slightly in the control group but significantly increased under 12 days of ABA treatment, rising from 27.4 to 105.4 U/g (**Figure 2D**). The pectate lyase (PL) activity increased and then decreased under ABA treatment, reaching a peak after 8 days, and showed no significant changes in the control group and NDGA treatment group (**Figure 2E**). Furthermore, the pectin methylesterase (PME) enzyme also peaked first after 8 days of ABA treatment (**Figure 2F**). ABA can regulate the content of pectin and improve the activity of pectin-degrading enzymes. In contrast, the NDGA treatment group played an inhibitory role in the change of pectin content.

### 3.3 Changes in volatile aromatic substances

**Figure 3:**
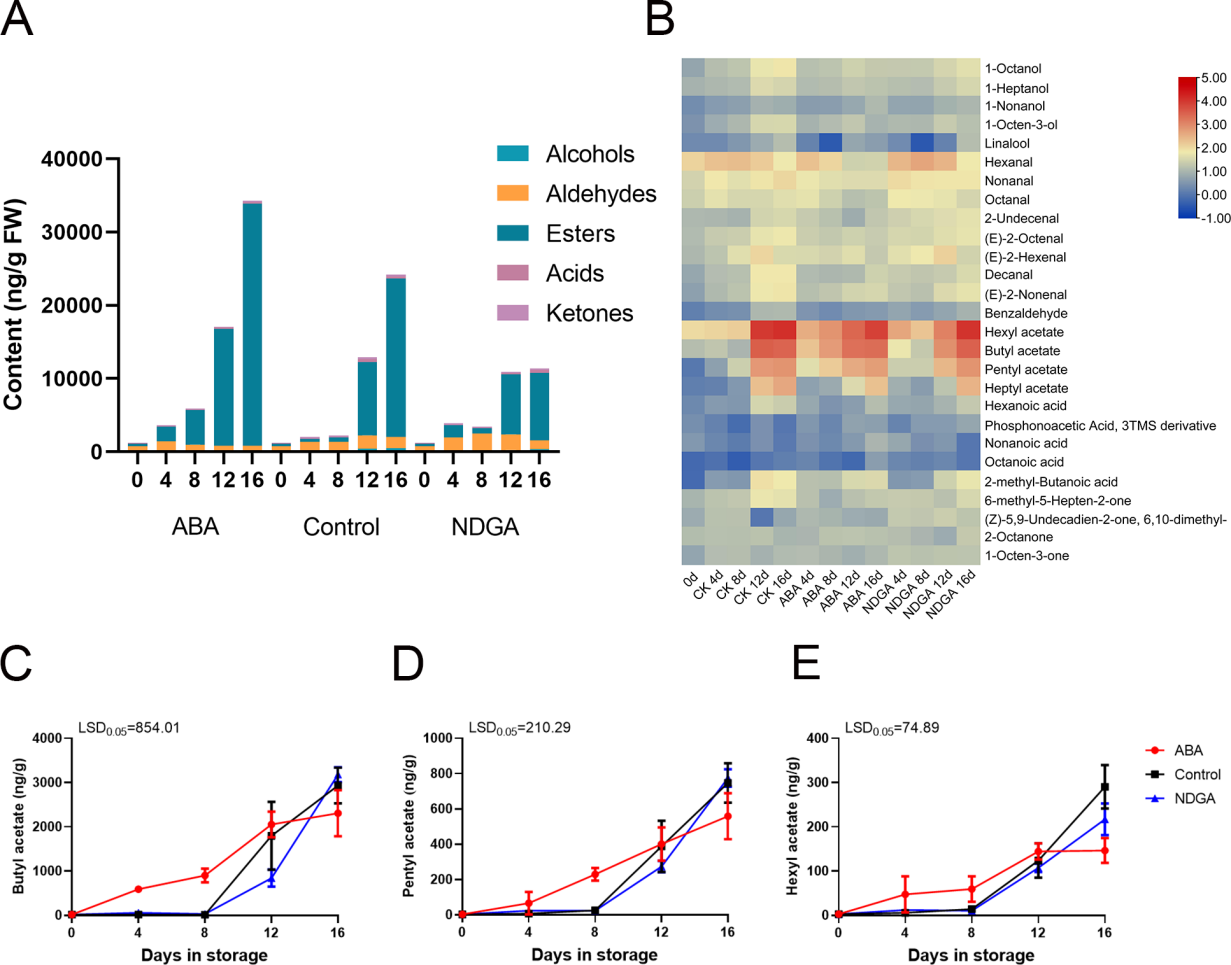
Volatile compound analysis of ‘Docteur Jules Guyot’ pear fruit. **(A)** Different volatile contents of ‘Docteur Jules Guyot’ pear fruit in ABA or NDGA treatment. **(B)** Heatmap presentation of all volatiles in ABA- or NDGA-treated pear fruit. **(C–E)** Profiles of acetic acid, butyl ester, acetic acid, pentyl ester, formic acid, and hexyl ester in the flesh of ‘Docteur Jules Guyot’ pear. The error bars come from three repeated SE. The observed LSD values suggest the presence of LSD with a significance level of *P* = 0.05.

The aroma compositions of ‘Docteur Jules Guyot’ pear under different treatments were determined, and it has been discovered that the fragrance of ‘Docteur Jules Guyot’ pear was composed of many substances (**Supplementary Table S2**). Fruits that produce volatile aromatic compounds can be categorized into five primary groups: alcohols, aldehydes, esters, acids, and ketones. Of these, esters undergo the most significant change in substance composition content. We found that the ester substance content was higher on day 4 in the fruit treated with ABA, and with the continuous ripening of fruits, the ester substance also gradually increased. Comparatively, the ester content of the NDGA treatment group was the lowest (**Figure 3A**). The heatmap representation of the various volatiles showed that esters have the highest substance content, with butyl acetate, amyl acetate, and hexyl formate being the most notable (**Figure 3B**). Under various treatments, the overall trend is increasing, with ABA treatment significantly increasing the ester substances. The initial levels of butyl acetate were 19.6 ng/g during storage and increased to 2,309.7 ng/g (**Figure 3C**), pentyl acetate increased to 558.3 ng/g (**Figure 3D**), and hexyl acetate increased to 146.4 from 3.2 ng/g (**Figure 3E**). This further demonstrates that the fragrance of ‘Docteur Jules Guyot’ pear during post-ripening is related to the increase of ester content. Among them, ABA treatment could promote the synthesis of ester substances, while NDGA treatment inhibited the synthesis of ester substances.

### 3.4 Expression of pectin degradation enzyme genes in different treatments

**Figure 4:**
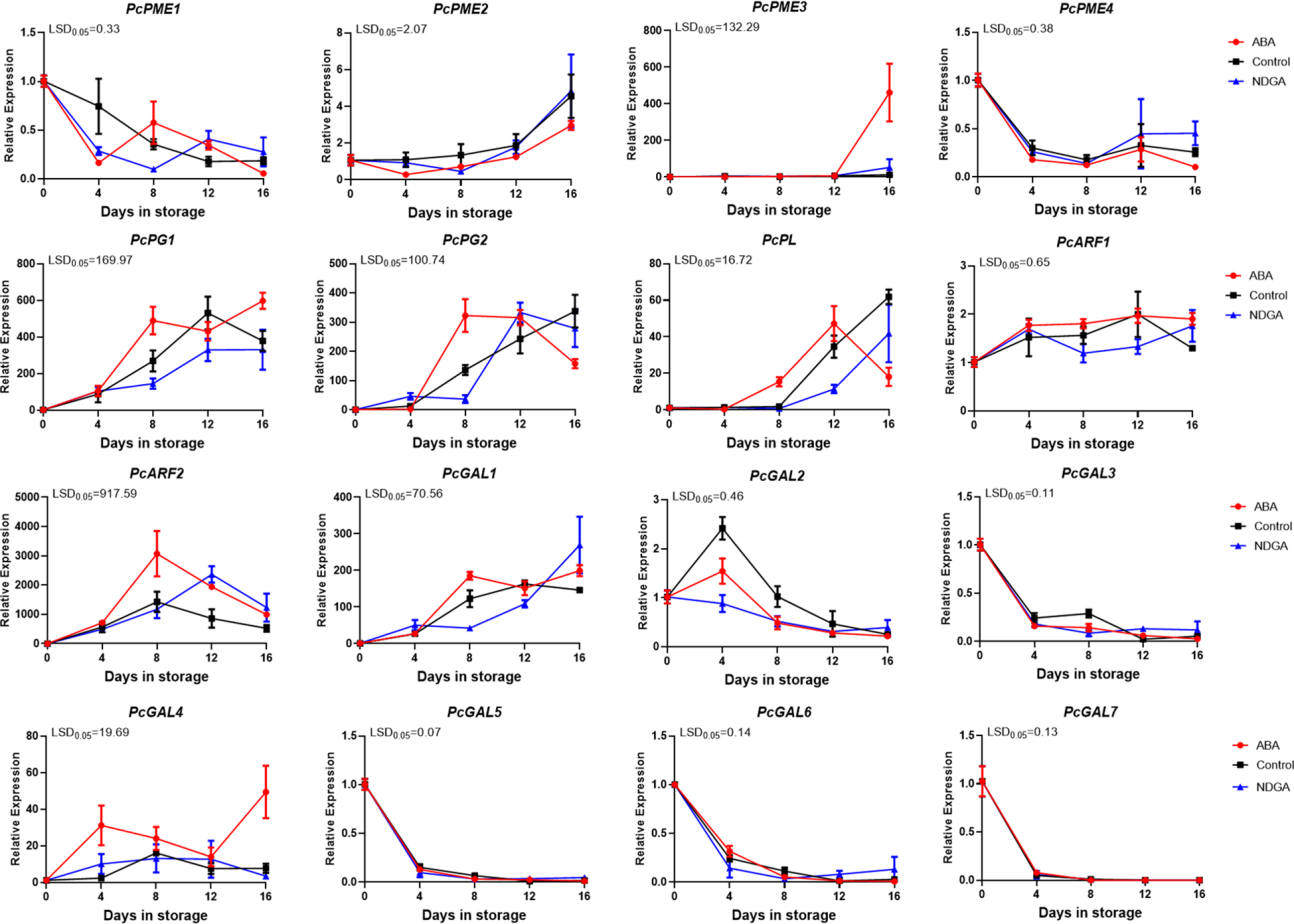
Expression of different pectin degradation enzyme encoding genes under ABA or NDGA treatment. The gene expressions of *PcPME*s, *PcPG*s, *PcPL*, *PcARF*s, and *PcGAL*s were analyzed using RT-qPCR. The error bars come from three repeated SE. The observed LSD values suggest the presence of LSD with a significance level of *P* = 0.05.

To verify the key genes affecting pectin degradation, we detected the expression of PG, PL, PME, β-galactosidase (β-Gal), and α-L-arabinofuranosidase (ARF) genes in different treatments (**Figure 4**). Before 12 days of various treatments, there was no significant difference in *PcPME3* expression. However, after 12 days, the ABA treatment group experienced a significant rise in *PcPME3* expression. Under ABA regulation, the expression of *PcPG1* and *PcPG2* gradually increased from day 4, peaked on day 8, and then decreased again. The expression of *PcPL* increased after 4 days under the ABA regulation and peaked after 12 days, while the *PcPL* expression in the control and NDGA treatment groups began to increase after 8 days. The expression of *PcARF2* first increased and then decreased, both the ABA treatment group and the control group peaked on the 8 days, the expression of the control treatment was lower than that of the ABA treatment group, and the peak was reached after 12 days in the NDGA treatment group. The expression of *PcGAL1* first peaked after 8 days under the regulation of ABA and increased slowly under the control group and NDGA treatment. The other genes showed no difference among ABA, NDGA, and control groups (**Figure 4**).

### 3.5 Expression of ABA biosynthesis genes in different treatments

**Figure 5:**
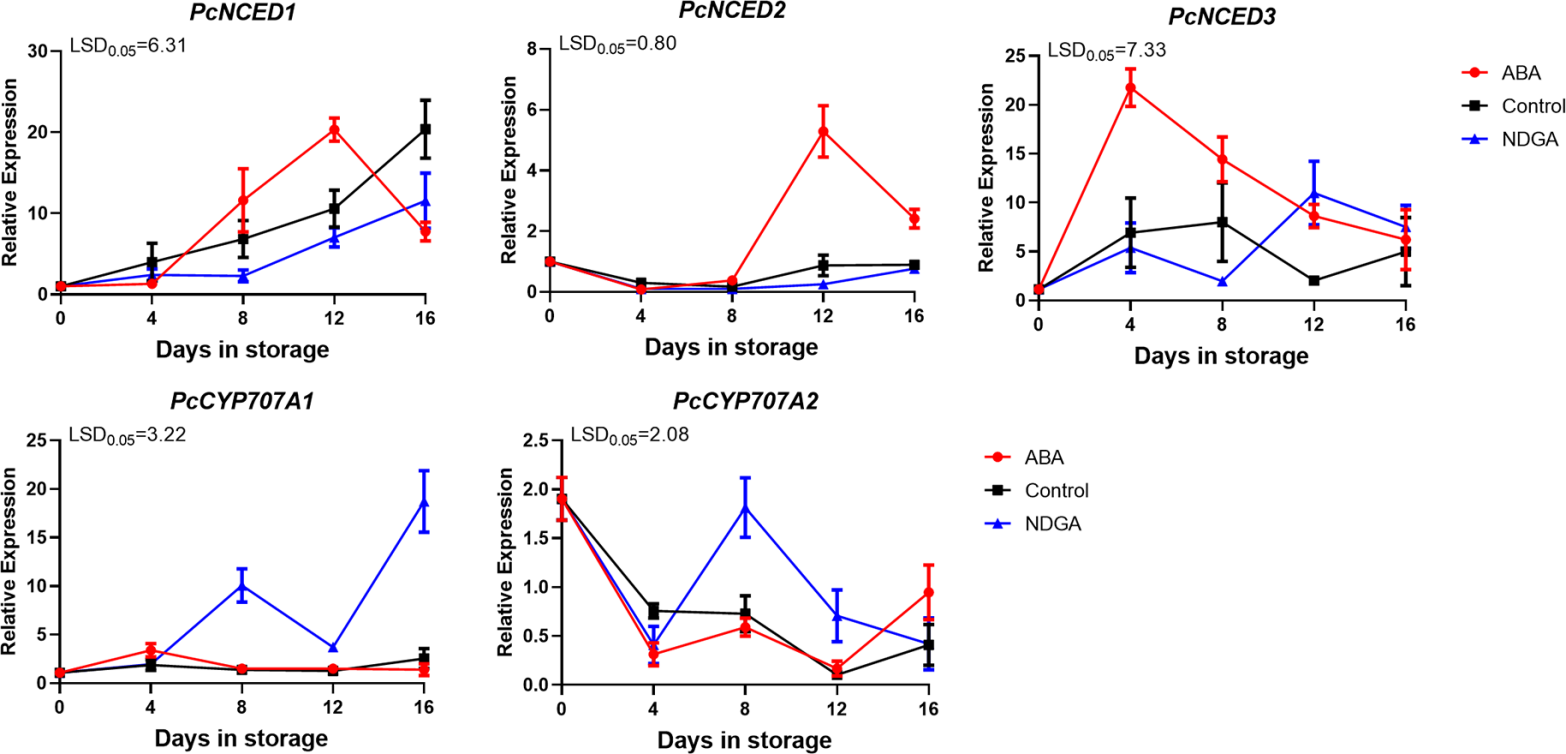
Expression of *PcNCED*s and *PcCYP707A*s under ABA or NDGA treatment. The error bars come from three repeated SE. The observed LSD values suggest the presence of LSD with a significance level of *P* = 0.05.

*PcNCED1* and *PcNCED2* showed an increasing trend under different treatments and first peaked after 12 days under ABA treatment. The trend was consistent between the control and NDGA treatment groups, and the expression levels were significantly lower than the ABA treatment group. The expression level of *PcCYP707A1* was significantly higher in the NDGA group, followed by the control, while the lowest was in the ABA treatment group (**Figure 5**).

### 3.6 Correlation between the physiological and biochemical indicators of ‘Docteur Jules Guyot’ pear under different treatments

**Figure 6:**
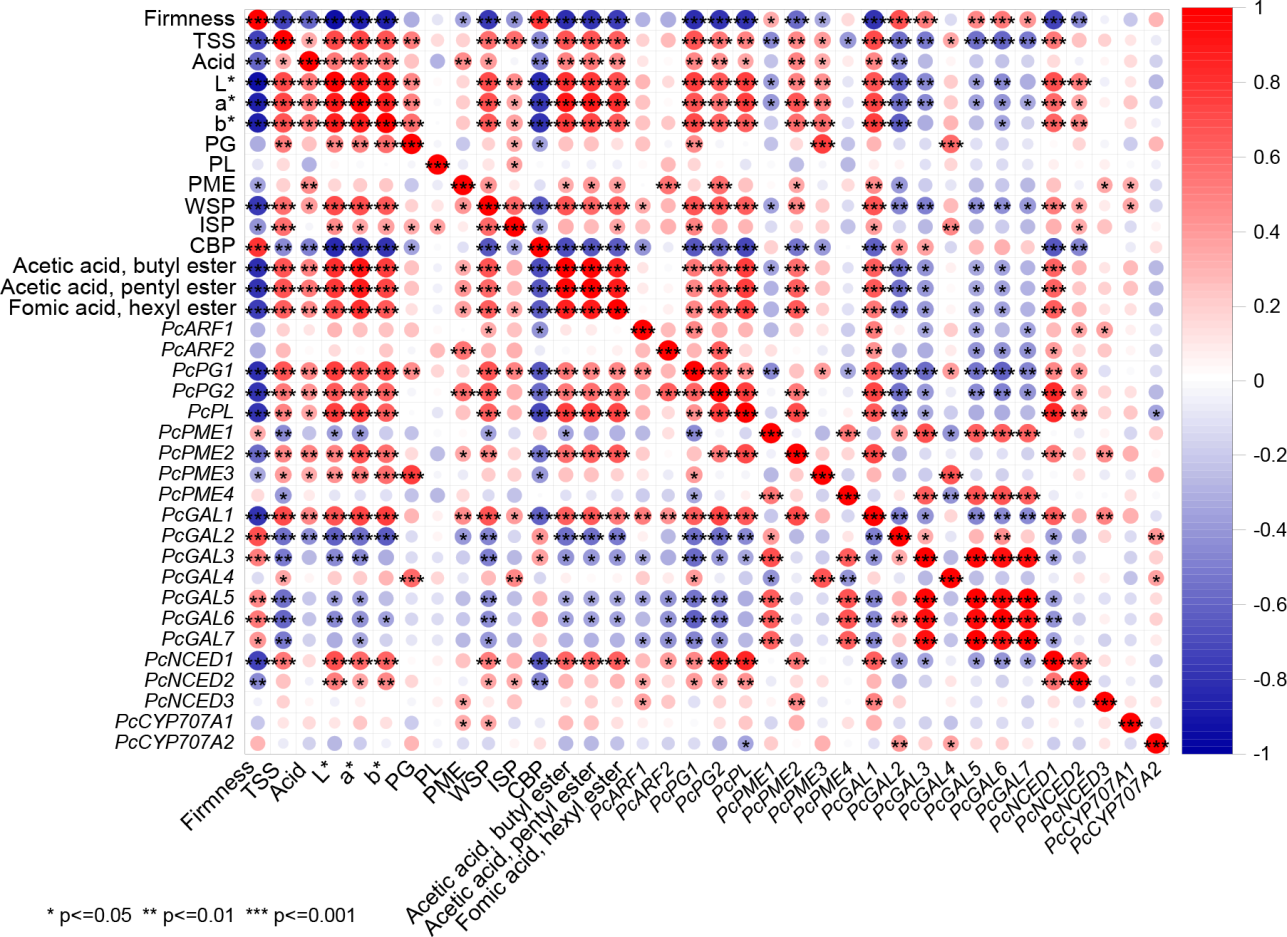
Correlation analysis between physiological properties, enzymatic activity involved in the degradation of pectin, pectin content, volatile compound contents, expression levels of pectin degradation-related enzyme genes, and the expression of *PcNCED*s and *PcCYP707A*s in ‘Docteur Jules Guyot’ pear fruit. Positive and negative correlations are shown in red and blue, respectively (*P < 0.05, **P < 0.01, ***P < 0.001).

By establishing a correlation analysis, it is possible to intuitively see the relationship between these physiological and biochemical indicators and the related genes of ‘Docteur Jules Guyot’ pear after harvest. Regarding physical characteristics, firmness was significantly negatively correlated with TSS, acid, L*, a*, b*, WSP, and ester compounds. In contrast, there were positive correlations between L*, a*, b*, TSS, acid, and WSP with esters. Moreover, there was a high positive correlation between CBP and firmness and a high negative correlation with L*, a*, and b*. In terms of related genes, apart from *PcPG1*, *PcPG2*, *PcPL*, *PcPME2*, *PcPME3*, *PcGAL1*, *PcNCED1*, and *PcNCED2*, the other genes exhibited a highly significant negative correlation (**Figure 6**).

## 4 Discussion

Research has shown that abscisic acid (ABA) can influence the epidermal color shift that occurs during fruit ripening in various fruit species. The changes in fruit epidermal color are closely correlated with the accumulation of anthocyanins. In climacteric fruit tomato, treatments with ABA and nordihydroguaiaretic acid (NDGA) also affected this process. The exogenous application of ABA resulted in an increase in ABA content, whereas NDGA inhibited this elevation. Compared to the control group, ABA treatment induced the coloration of tomato, while NDGA treatment delayed the coloring process (Mou et al., 2016). In the non-climacteric fruit strawberry, ABA promotes fruit ripening by reducing the time required to reach the fully red stage (Lu et al., 2023). Furthermore, the *h* value of citrus fruit color index indicated that exogenous ABA can accelerate the coloring of citrus fruit, while NDGA treatment can inhibit fruit coloring (Wang et al., 2016). This study investigated the application of ABA, which accelerated pear fruit coloration by 4 days over the control group. This indicates that ABA can influence the epidermal color change in pear fruit (**Figure 1**).

The degree of connection between cell walls will ultimately affect the texture of the fruit. During the maturation process, changes in pectin, hemicellulose, and cellulose are considered to be the reasons for the changes in cell wall structure during the period of firmness loss related to maturity (Chen et al., 2015). Research shows that water-soluble pectin (WSP) increases, indicating that the change of pectin was related to the long-term stored quality of fruit (Murayama et al., 2006). The modifications of the fruit cell wall vary according to species (Forlani et al., 2019). For some climacteric fruits such as kiwifruit, tomato, and avocado, pectin is depolymerized during fruit ripening, whereas in the climacteric fruit apple and non-climacteric fruit watermelon, pectin solubilization and depolymerization have a low degree of pulp-melted fruit, indicating that the firmness of the fruit depends on the integrity of the cellular connections (Brummell, 2006). After analyzing the composition of cell wall components in ‘Docteur Jules Guyot’ pear, it was observed that there was an increase in the levels of WSP and ionic-soluble pectin (ISP) content, and the content changes were the largest under the promotion of ABA (**Figure 2**).

The associated metabolic enzymes can affect the texture of fruit through the cell wall. It has previously been reported that polygalacturonase (PG) is an essential factor related to the change of pectin components (Wakabayashi, 2000). PG activity and cell wall modification in hard-tasting fruits are lower than softer fruits. After *MdPG1* was silenced, the fruit softening was slower, and the pectin content was reduced (Atkinson et al., 2012). Research has found that the transcriptional accumulation of the PG gene was well correlated with softening in pear fruit (Sekine et al., 2006). Furthermore, pectate lyase (PL) activity appears to be more widely recognized in ripe fruits (Al Hinai et al., 2021). The findings indicated that during the ripening and softening of the ‘Docteur Jules Guyot’ pear, the activities of PG and PL under ABA treatment remained high, contrasting with the fruit firmness trend (**Figure 2**).

Fruit aroma can reflect the flavor quality of fresh fruit and has become an important factor influencing consumer choice. Fruit flavors are made up of a complex mixture of substances, and the level of different substances affects the variation of fruit flavors. It has been found that, in most fruits, volatile compounds consist mainly of esters, alcohols, aldehydes, and ketones (El Hadi et al., 2013). During the ripening of ‘Pink Lady’ apples, esters such as hexyl acetate, hexyl 2-methylbutyrate, hexyl caproate, and hexyl butyrate accounted for a relatively large proportion of the fruit volatiles (Villatoro et al., 2008). In European pear, esters have also been identified as the main volatile compound substances (Rapparini and Predieri, 2002). From our experimental results, we can see that the ester content of the fruits increased significantly after ABA treatment (**Figure 3**). Previous studies had also found that ABA can promote the production of short-chain ester compounds in apple (Wang et al., 2018). In addition, ABA treatment also significantly increased the production of esters in fruit aroma in low-temperature-treated kiwifruit (Han et al., 2022). Therefore, ABA treatment not only promoted the softening of the pear fruit but also promoted the pear fruit to emit a stronger fruit flavor.

In European pear, the expression of genes associated with cell walls during the softening process was also studied (Fonseca et al., 2005). In non-climacteric fruit strawberry, silencing both of the PL gene *FaPLC* and the PG gene *FaPG1* slowed the softening of the fruit (Jiménez-Bermúdez et al., 2002; Quesada et al., 2009). Similarly, we found that under the induction of ABA, the expression of *PcPG1*, *PcPG2*, and *PcGAL1* increased quickly (**Figure 4**). 9-cis-Epoxy carotenoid dioxygenase (NCED) plays a key role in ABA synthesis and has been isolated from various fruits (Burbidge et al., 1999). At the transcriptional level, the ABA content in climacteric fruit tomato was regulated by *SlNCED1* (Zhang et al., 2009a). ABA accumulation was enhanced in *SlNCED1* over*-*expressed in tomato (Ji et al., 2014). After silencing *SlNCED1* in transgenic tomato, endogenous ABA content decreased and cell wall depolymerization degree and fruit ripening were also inhibited (Thompson et al., 2000). The results showed that during the ripening process of ‘Docteur Jules Guyot’ pear, under the regulation of ABA, the gene expression of *PcNCED1* and *PcNCED2* was upregulated (**Figure 5**). It has been found that ABA promotes fruit ripening through a signaling cascade pathway (Leng et al., 2013). It has been predicted that NAC TFs can directly target genes encoding enzymes that catalyze changes associated with degradation of cell wall components or lignification pathways, indirectly affecting fruit texture by regulating endogenous ABA and ethylene levels, which promotes the expression of downstream genes (Liu et al., 2022). In tomato fruit, SlNOR promotes cell wall degradation by directly binding to the promoter region of the cell wall degradation-related gene *SlPL* and activating its expression (Gao et al., 2020). SlNOR-like 1 promotes tomato fruit softening by activating *SlPG2a*, *SlPL*, *SlCEL2*, and *SlEXP1* (Gao et al., 2018).

## 5 Conclusion

The post-harvest ‘Docteur Jules Guyot’ pear was treated with abscisic acid (ABA) and nordihydroguaiaretic acid (NDGA), and the results showed that ABA treatment accelerated the reduction of fruit firmness. The changes in the contents of water-soluble pectin (WSP), ionic-soluble pectin (ISP), and covalent binding pectin (CBP) destroyed the ligation properties in the cell wall and improved the activity of related metabolic enzymes. The volatile ester content associated with pear fruits increased substantially. On the other hand, NDGA treatment delayed the softening rate of the fruit, and the content of ester compounds in volatile aromatics was also low. Meanwhile, the activity of pectin degradation-related enzymes was reduced. In addition, ABA treatment also upregulated the essential pectin degradation enzyme genes *PcPG1*, *PcPG2*, *PcPL*, *PcPME2*, *PcPME3*, and *PcGAL1* and the ABA biosynthetic encoding genes *PcNCED1* and *PcNCED2*. In summary, the results showed that ABA treatment could shorten the post-ripening and softening period of ‘Docteur Jules Guyot’ pear fruit and advance the eating time of the fruit.

## Data Availability

The original contributions presented in the study are included in the article/**Supplementary Material**, further inquiries can be directed to the corresponding author.

## Supplementary material

Supplementary Table 2 Overview of the GC-MS results.
