# Supplementary material for: Mechanism of abscisic acid in promoting softening of postharvest ‘Docteur Jules Guyot’ pear (Pyrus communis L.)
Source: Front Plant Sci. 2024 Dec 17;15:1502623. doi: 10.3389/fpls.2024.1502623 (PMC11685007; doi:10.3389/fpls.2024.1502623)
Supplement: Supplementary file 2 [file Table1.docx]

**Supplementary**

Table S1 Primers sequences used in this study.

| Gene Name | Primer Sequence (5’-3’) |
| --- | --- |
| *PcACTIN*-F | TTTATGCCAGTGGTCGTA |
| *PcACTIN*-R | TCCCGTTCAGCAGTAGTG |
| *PcPME1*-F | TCGAGGGTTCAAAGGGTAGC |
| *PcPME1*-R | AGGTAATAACCCGATACCCAGG |
| *PcPME2*-F | ACCATCCTTGTTCGTCCTAGAC |
| *PcPME2*-R | GTCTCCCCAGGTAGTTCTTGTG |
| *PcPME3*-F | ACCAACCACCAAACTTGCTC |
| *PcPME3*-R | TTTGTCCTTAGGCACCCAAC |
| *PcPME4*-F | TCCTTGAGGGACACCATTGG |
| *PcPME4*-R | AACTTAGTTTCCCTGTGCTGT |
| *PcPG1*-F | ATGGCTTTAAAAACACAGTTG |
| *PcPG1*-R | ACCTTCAATGGTGTCCATGTAT |
| *PcPG2*-F | CTAATGACAAAGGTTGCCCTGG |
| *PcPG2*-R | AGACAACTTGTAGGCTGAACC |
| *PcPL*-F | ATCCACGGACTGCACATACA |
| *PcPL*-R | ACCCATCTTTGCAGTTGGAC |
| *PcARF1*-F | GGACTGACGATGGACTTGGT |
| *PcARF1*-R | TACCGTCGAGAGCTTCCTGT |
| *PcARF2*-F | GCGAGGTACACAAGGACCAT |
| *PcARF2*-R | GTCCGGTCTCTGAACTCTGC |
| *PcGAL1*-F | GACTGGTCTGAAAGGCGAAG |
| *PcGAL1*-R | CCCACGCTCTGTCCATTTAT |
| *PcGAL2*-F | CCACCTGTCCAGAAGTGGTT |
| *PcGAL2*-R | TGGAGACAATGGACGATGAA |
| *PcGAL3*-F | GCTAGTTCAAACGGCGAAAG |
| *PcGAL3*-R | GGCCAATTCGAAGAATCAAA |
| *PcGAL4*-F | AAAGGCAATCGCTTTTGAGA |
| *PcGAL4*-R | TGGCCTTCTGAATCAAATCC |
| *PcGAL5*-F | ATTGCACTGCTGAGTGTTGC |
| *PcGAL5*-R | ATGGCTGCTGCTTCTTTTGT |
| *PcGAL6*-F | ATCAGGTGCCGGAATAACTG |
| *PcGAL6*-R | ACAGGGTTACTGCCATCAGG |
| *PcGAL7*-F | ACTTGGAGCTCCTGGACAGA |
| *PcGAL7*-R | CAGGTCGATGGTGAATTGTG |
| *PcNCED1-F* | AAGGAAGCCACTCGAGCAAA |
| *PcNCED1-R* | TTTGGACACGTGGGTCTGAG |
| *PcNCED2-F* | GATCGTGGTTATCGGCTCGT |
| *PcNCED2-R* | TTCTCCCTAGCTTGTCCCGA |
| *PcNCED3-F* | CATGGACCCACCGGATTCAA |
| *PcNCED3-R* | AGCAATACCCGAACACCTGG |
| *PcCYP707A1-F* | CTTCCCTCAGCCCGAAAAGT |
| *PcCYP707A1-R* | AAGCAGTGGTGAGGTGATGG |
| *PcCYP707A2-F* | ATCTTCCAGGCACGCTGTTT |
| *PcCYP707A2-R* | GATGACCCCAATGACGTTGTC |
